# Antibacterial activity of exopolysaccharide produced by bee gut-resident *Enterococcus* sp. BE11 against marine fish pathogens

**DOI:** 10.1186/s12866-023-02977-9

**Published:** 2023-08-23

**Authors:** Eman H. Zaghloul, Mohamed I. A. Ibrahim, Heba A. H. Zaghloul

**Affiliations:** 1 National Institute of Oceanography and Fisheries (NIOF), Cairo, Egypt; 2 Department of Botany and Microbiology, Faculty of Science, Alexandria University, Moharam Bek 21511, Alexandria, Egypt

**Keywords:** Lactic acid bacteria, Exopolysaccharides, Fish pathogens, *Enterococcus* sp, Bees

## Abstract

**Background:**

In recent years, the demand for innovative antimicrobial agents has grown, considering the growing problem of antibiotic resistance in aquaculture. Adult *Apis mellifera* honeybees’ gut represents an outstanding habitat to isolate novel lactic acid bacteria (LAB) able to produce prominent antimicrobial agents.

**Methods:**

In the current study, twelve LAB were isolated and purified from the gut of adult *Apis mellifera.* The isolates were screened for exopolysaccharide (EPS) production. The most promising isolate BE11 was identified biochemically and molecularly using 16 S rRNA gene sequence analysis as *Enterococcus* sp. BE11 was used for the mass production of EPS. The partially purified BE11-EPS features were disclosed by its physicochemical characterization. Moreover, the antimicrobial activity of BE11 cell free supernatant (CFS) and its EPS was investigated against some fish pathogens namely, *Pseudomonas fluorescens*, *Streptococcus agalactiae*, *Aeromonas hydrophila*, *Vibrio* sp. and *Staphylococcus epidermidis* using well-cut diffusion method.

**Results:**

The physicochemical characterization of BE11-EPS revealed that the total carbohydrate content was estimated to be ~ 87%. FTIR and NMR analysis ascertained the presence of galactose and glucose residues in the EPS backbone. Moreover, the GC-MS analysis verified the heterogeneous nature of the produced BE11-EPS made up of different monosaccharide moieties: galactose, rhamnose, glucose, arabinose sugar derivatives, and glucuronic acid. BE11 CFS and its EPS showed promising antimicrobial activity against tested pathogens as the inhibition zone diameters (cm) ranged from 1.3 to 1.7 and 1.2–1.8, respectively.

**Conclusion:**

The bee gut-resident *Enterococcus* sp. BE11, CFS, and EPS were found to be promising antimicrobial agents against fish pathogens and biofilm producers affecting aquaculture. To the best of our knowledge, this is the first study to purify and make a chemical profile of an EPS produced by a member of the bee gut microbiota as a potential inhibitor for fish pathogens.

## Introduction

Lactic acid bacteria (LAB) produce exopolysaccharides (EPS) that are chemically diverse and can be homopolysaccharides or heteropolysaccharides. The liberated EPS is either bound to the cell wall as a capsule or released into the medium as a sticky slime [1–3]. LAB and their EPS are known for their significant biotechnological and health contributions. In the fermented food industry, for example, EPS can compensate for the lack of specific food ingredients and provide better end-product qualities. Furthermore, concerning human health-related applications, several EPS were found to possess anticancer, antitumor, immunomodulation, cholesterol-lowering, and prebiotic properties [4].

Although research has limited the use of LAB and its EPS in the fields related to food and health, other biotechnological applications are yet to be explored. For example, the antibiofilm activities of some LAB isolates [5] or their EPS [6] are impressive and can help to protect maritime ecosystems from biofilm complications. Biofilm production contributes to marine equipment micro- and macro-fouling [7], biocorrosion [8], and aquaculture collapse [9]. Many fish pathogens are also biofilm producers in the marine environment for example, *Vibrio* sp., *Staphylococcus* sp., *Streptococcus* sp., *Pseudomonas* sp., and *Aeromonas* sp [10–13]. All these pathogens are known to cause devastating diseases in the infected host. For example, *Pseudomonas fluorescens* and *Aeromonas hydrophila* are known as opportunistic pathogens found in the aquatic environment and healthy fish gut flora. They pose a high risk upon spread in aquatic systems for fish that are highly sensitive to water pollution [14]. Marine fish species that are affected by *A. hydrophila* include Meagre, Grouper, Sea bream, and Mullet [15]. Both *Pseudomonas fluorescens* and *Aeromonas hydrophila* are associated with hemorrhagic septicemia in marine fish such as *Tilapia zillii* and *Mugil capito* [16–18]. *Staphylococcus epidermidis*, isolated previously from marine habitats [19], is also known to induce septicemia along with exophthalmia, congestion, and ulcers development in fishtails [20, 21]. *Streptococcus agalactiae* are associated with bacteremia and meningitis induction if infected fish are consumed by humans [22]. Moreover, it causes significant loss in the essential commercial tilapia aquaculture [23]. *Vibrio* sp. is the causative agent of vibriosis, a disease that negatively affects fish, crabs, shrimp, and lobsters. Therefore, preventive measures are essential to avoid outbreaks of this pathogen [24].

Because bacteria may form biofilms and persist in water even when they are not associated with the host, aquaculture has become particularly vulnerable to bacterial diseases [25]. Therefore, identifying novel LAB isolates and EPS effective against these fish pathogens and biofilm producers is thus viewed as a line of defense against these detrimental effects on the marine environment. Antimicrobial resistance deaths are currently estimated to be 700,000 per year, with the number expected to increase to 10 million by 2050. Therefore, the necessity to discover new natural antimicrobial drugs has emerged in recent years considering the increasing antibiotic resistance dilemma in aquaculture because of the widespread and uncontrolled use of antibiotics [26, 27].

LAB capable of EPS production are common residents in a variety of habitats. For example, they can be isolated from human and other vertebrate digestive tracts, fermented food, fruits, and flowers [28, 29]. LAB genomes contain a large number of metabolic genes that allow them to grow in this wide range of conditions [30]. The gut environment of honeybee *Apis mellifera* is one of the LAB habitats that are far from being fully understood. The gut of bees is the principal site of digestion, food processing, and occasional pathogens attack [31, 32]. As a result, it is hypothesized that bee gut LAB isolates and their EPS may have novel antimicrobial properties against pathogens and thus contribute to the reduction of aquaculture antibiotic resistance.

Therefore, the current study aimed to investigate the activity of an *Apis mellifera* gut-resident *Enterococcus sp.* (BE11) and its EPS against important fish pathogens and biofilm producers, namely *Vibrio sp., Pseudomonas fluorescens*, *Streptococcus agalactiae*, *Aeromonas hydrophila and Staphylococcus epidermidis*. Furthermore, physicochemical characterization of BE11-EPS using a UV-visible spectrophotometer, Fourier transform infrared spectroscopy (FTIR), nuclear magnetic resonance (NMR), gas chromatography-mass spectrometry (GC-MS), scanning electron microscope (SEM), and energy dispersive X-ray analysis (EDX).

## Materials and methods

### Bees collection and LAB isolation

The bee *Apis melifera* adults were collected from Kafr El-Dwar governorate, Egypt. For LAB isolation, each insect was surface sterilized using 96% ethanol and dissected on the surface of a sterilized wax plate to separate the insect’s gut. The separated gut was kept in de MAN, Rogosa and Sharpe (MRS) broth (HiMedia) for enrichment at 37 °C for 24 h under anaerobic conditions in a candle jar [33]. For purification, each liquid culture (6 cultures) was serially diluted and plated on the surface of MRS agar under the same incubation conditions for 24–48 h. Finally, 12 individual phenotypically unique colonies were picked for further purification using 2–3 rounds of the streak-plate method.

### Screening, production, and purification of EPS from LAB strains

**Fig. 1:**
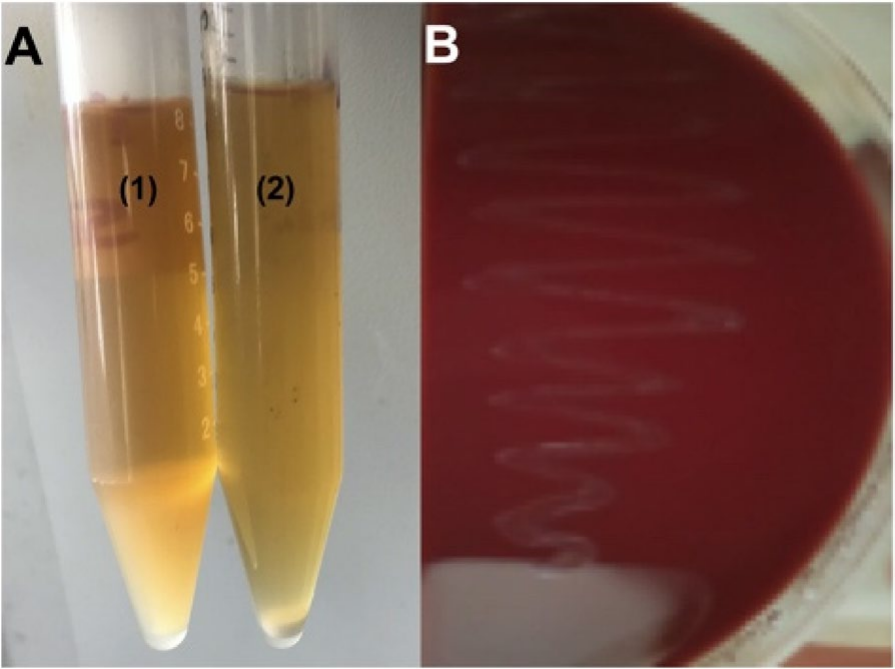
Growth of *Enterococcus* sp. BE11 isolate on liquid MRS and blood agar. **A** accumulation of BE11 isolate exopolysaccharide slime in liquid MRS supplemented with 1.0% sucrose sugar (1) versus BE11 growth in absence of sucrose (2). **B** gamma-hemolysis (no hemolysis) of BE11 cells on the surface of blood agar after incubation for 24 h at 37° C

The twelve bacterial isolates were screened for EPS production on the surface of MRS agar plates supplemented with 1.0% sucrose sugar for 24 h at 37 °C. Isolate BE11 showed a mucoid ruby appearance. Moreover, it showed no signs of hemolysis on the surface of blood agar (Fig. 1), therefore, it was selected for further study. For EPS production, the BE11 isolate was grown in one liter MRS broth supplemented with 1.0% sucrose sugar for 48 h at 37 °C. For the removal of bacterial cells, the bacterial culture was centrifuged at 2000 × g for 10 min at 4 °C. The supernatant was then filtered using a bacterial filter (pore size 0.45 µM). For protein degradation, the filtrate was exposed to 10% w/v trichloroacetic acid (TCA) for 30 min. The degraded protein was collected by centrifugation at 2000 × g for 15 min at 4 °C. Absolute ice-cold ethanol was added to the protein-free supernatant in a 3:1 (v/v) ratio and stored for 2 days at 4 °C. Finally, the precipitate collected after centrifugation at 2000 × g for 15 min at 4 °C was recovered and dialyzed against distilled water at 30° C and the dry weight of the obtained EPS was detected.

### Phenotypic and biochemical characterization of isolate BE11

The pure culture of BE11 was examined morphologically to determine the cells’ Gram reaction and cell shape. A scanning electron microscope (SEM) was used to observe the characteristic cell shape and size. For biochemical characterization, the VITEK system (BioMérieux, France) was used (available through Mabaret El-Asafra Hospital, Alexandria, Egypt). The VITEK is a fully automated system for bacterial cell identification. It examines the cells for a series of biochemical tests such as sugar fermentation, enzyme hydrolysis, and antibiotic resistance.

### The 16 S rRNA gene amplification and sequencing

To precisely identify the genus and/or bacterial species of the purified bacterial strain, the bacterial DNA was isolated following the instructions of the DNA isolation kit (Qiagen, Germany) followed by PCR amplification, purification, and sequencing through Applied Biotechnology co., Egypt. The 16 S rRNA gene was amplified using the following primer pairs: Uni27F, 5′-AGAGTTTGATCCTGGCTCAG-3′ and Uni1492R, 5′-GGTTACCTTGTTACGACTT-3′ [34]. The PCR program was adjusted as follows: 3 min at 94 °C, followed by 35 cycles at 94° C for 30 s, 55° C for 30 s, and 72° C for 1 min. Finally, the reaction was kept at 72° C for 10 min. The quality of the resulting Sanger sequence chromatogram was confirmed before being submitted to GenBank (NCBI) under the accession number MW218439.1.

### Phylogenetic analysis

BLASTn homology search revealed that isolate BE11 showed 99.71% sequence similarity to *Entercoccus lactis* strain BML2 (NCBI accession No. MN560018.1). The partial sequence of the 16 S rRNA gene was aligned against 14 *Enterococcus* species using the default ClustalW parameters. The aligned sequences were used for phylogenetic tree construction. The Maximum Likelihood method combined with the bootstrapping of 100 replicates available through MEGA7 software was used for tree construction [35, 36]. As an outer group, the *Pediococcus acidilactici* strain NGRI 0510Q was included (NCBI accession number NR 041640.1).

### Physicochemical characterization of BE11-EPS

#### Total carbohydrate measurement by UV-visible spectrometry

Estimation of the total carbohydrate content in the partially purified BE11-EPS was made using the phenol-sulfuric acid method [37]. In brief, to 1.0 mL aliquot of the purified BE11-EPS, about 1.0 mL phenol (2.5%; w/v) and 5.0 mL H_2_SO_4_ (purity > 98%) were added consecutively. Then, the mixture was vortexed and incubated at ambient temperature for 20 min before recording the absorbance at 480 nm against a blank reagent using a UV-Visible spectrophotometer (JENWAY 6800, UK). The carbohydrate content was expressed as glucose%, compared to the glucose standard curve.

#### Structural study by FTIR

The chemical functionalities of the partially purified BE11-EPS were identified using FTIR spectrometer (Bruker, ALPHA, Germany) equipped with the attenuated total reflectance (ATR) technique. About 2.0 mg of the dried extract was loaded over a germanium crystal, and the spectrum was acquired between 4000 and 400 cm^-1^ with a resolution of 4.0 cm^-1^, after subtracting the atmospheric background interference.

#### Structural analysis by NMR

The nature of BE11-EPS was investigated using NMR analysis. About 10 mg of the dried sample was dissolved in 0.5 mL of DMSO-*d*_6_, then the solution was gently warmed for complete dissolution. Then, the ^1^ H NMR spectrum was recorded using JEOL-Ltd spectrometer (500 MHz, Japan) at 323 K. The chemical shifts are expressed in ppm (δ) relative to tetramethylsilane (TMS) as an internal standard (δ = 0 ppm).

#### Monosaccharide identification by GC-MS

The sugar constituents of BE11-EPS were identified as the silylated glycosides through acid hydrolysis step, silylation derivatization step followed by GC-MS analysis [38–41]. Acid hydrolysis was performed by treating 20 mg of the dry sample with 3.0 mL of sulfuric acid (2 M) in a sealed glass tube. The mixture was heated to 105° C for 10 h for complete hydrolysis. The tube was cooled at RT before neutralization with barium carbonate (pH 7.0). The precipitate formed was then removed by centrifugation, while the supernatant was filtered through a 20 μm syringe before lyophilization. Subsequently, dried hydrolysates were derivatized by silylating with 1:1 pyridine-BSTFA (*N,O*-bis(trimethylsilyl)trifluoroacetamide) for 16 h at 80 °C, using 50 µL per mg of the dried sample [39, 41]. Finally, 2.0 µL of the silylated sugars were injected into GC-MS (MassHunter GC-MS 1989–2014, Agilent Technologies, Inc.). The method of separation and detection of the monosaccharides were done following a previously reported method: the column used was HP5MS (30 m × 0.25 mm × 0.25 μm), the temperatures of the detector and the injector were set at 320 °C, the column temperature was firstly set at 100 °C for 1.0 min, and then ramped from 100 to 260 °C at 4 °C min^−1^, then the temperature was set for 10 min at 260 °C. Helium was used as carrier gas at 1.0 mL min^−1^ [42]. Identification of monosaccharide derivatives was carried out by comparing the recorded mass spectra with the NIST [43].

#### Morphological and elemental studies by SEM and EDX spectroscopy

The morphological inspection of the dried BE11-EPS was visualized using scanning electron microscopy (SEM, JSM-IT 200, Jeol, Japan). On the other hand, the elemental composition of BE11-EPS was investigated using a scanning electron microscope energy dispersive x-ray (SEM-EDX) spectrometer.

#### Anti-fish pathogens activity of BE11 CFS and EPS

The antibacterial activity of BE11 CFS was tested against a set of fish pathogens and biofilm producers, namely, *Vibrio* sp., *Pseudomonas fluorescens*, *Streptococcus agalactiae*, *Staphylococcus epidermidis* and *Aeromonas hydrophila*. The agar well-cut diffusion method was used for this analysis. **First**, BE11 CFS activity was tested against each pathogen. Briefly, the BE11 isolate was cultivated overnight at 37 °C on MRS broth supplemented with 1.0% sucrose sugar, then centrifuged for 15 min at 4 °C at 2000 x g and the pH was adjusted to 6.5 by 1 N NaOH. Finally, a bacterial filter (pore size 0.45 µM) was used to filter the collected supernatant. Only 100 µL of filtered supernatant was used to fill a well on the surface of a nutrient agar plate that had been inoculated with 10^6^ CFU/mL of the fish pathogen under investigation. Sterile MRS broth was used as negative control and Vancomycin_30_ (Oxoid, USA) was used as positive control **Second**, BE11-EPS was examined against each pathogen. Briefly, 100 mg of EPS was dissolved in 1.0 mL of dimethylsulfoxide (DMSO). Only 100 µL of EPS dissolved in DMSO solution was used to fill each well on the surface of the nutrient agar plate inoculated by 10^6^ CFU/mL of the fish pathogen under test. DMSO served as a negative control and Vancomycin_30_ (Oxoid, USA) served as a positive control [44].

### Statistical analysis

Experiments were conducted in triplicate and results were expressed as means ± standard deviation (SD). Statistical analysis (paired t-test) was performed using GraphPad software (Prism9).

## Results

### Growth of isolate BE11 on the surface of different media

The growth of isolate BE11 was tested on the surface of two media. First, the growth on the surface of the blood agar did not show signs of hemolysis (Fig. 1). Second, the growth of BE11 on the surface of MRS agar supplemented with 1.0% sucrose sugar resulted in the formation of mucoid and ruby colonies, implying the production of exopolysaccharides [45]. Specifically, BE11 isolate produced 173 mg/L EPS in MRS broth supplemented with 1.0% sucrose sugar.

### Morphological, biochemical, and molecular characterization of BE11 isolate

**Fig. 2:**
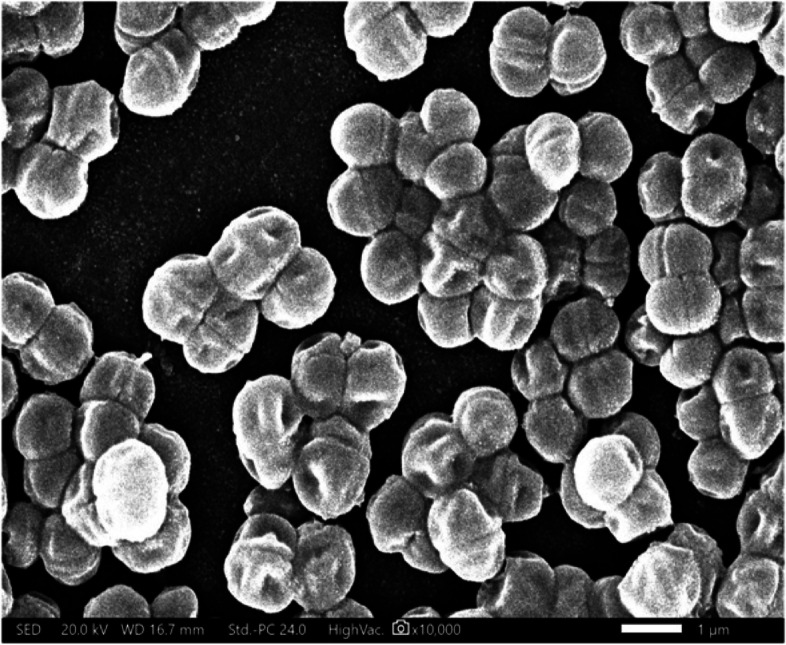
Scanning electron microscopy (SEM) imaging of *Enterococcus* sp. BE11 bacterial cells. It demonstrates the BE11 characteristic spherical cell shape, size, and grouping in clusters using SEM

The bacterial isolate BE11 was identified as Gram-positive and catalase-negative cocci. Furthermore, the scanning electron microscope (SEM) examination of cells revealed their characteristic spherical cell shape and cell grouping in clusters (Fig. 2). The VITEK2 microbial identification system (BioMérieux, France) was employed for biochemical characterization. BE11 isolate was identified as *Pediococcus pentosaceus* (98% probability) (Table 1). The molecular identification based on the 16 S rRNA gene sequence does not match this finding. BE11 isolate was identified as *Enterococcus lactis* species (99.71% identity) based on the 16 S rRNA gene sequence. The gene sequence was deposited in the GenBank database of the National Center for Biotechnology Information (NCBI) with the accession number MW218439.1. The VITEK2 method, on the other hand, revealed certain key biochemical properties for BE11 isolate, including resistance to three antibiotics, Bacitracin, Novobiocin, and Optochin, as well as the ability to grow in 6.5% NaCl (Table 1). The growth in high salt concentrations suggests that BE11 could be used in aquatic marine cultures and a variety of high-salt industries.

**Table 1:** Biochemical characterization of BE11 bacterial isolate using the VITEK system (BioMérieux, France)

Test	Result	Test	Result
D-amygdalin (AMY)	+	D-galactose (dGAL)	+
Phosphatidylinositol phospholipase c (PIPLC)	-	D-ribose (dRIB)	-
D-xylose (dXYL)	-	L-lactate alkalinization (ILATK)	-
Arginine dihydrolase 1 (ADH1)	+	Lactose (LAC)	-
Beta-galactosidase (BGAL)	-	N-acetyl-d-glucosamine (NAG)	+
Alpha-glucosidase (AGLU)	-	D-maltose (dMAL)	+
Ala-phe-pro arylamidase (APPA)	-	Bacitracin resistance (BACI)	+
Cyclodextrin (CDEX)	-	Novobiocin resistance (NOVO)	+
L-aspartate arylamidase (AspA)	-	Growth in 6.5% NaCl (NC6.5)	+
Beta galactopyranosidase (BGAR)	-	D-mannitol (dMAN)	-
Alpha-mannosidase (AMAN)	-	D-mannosE (dMNE)	+
Phosphatase (PHOS)	-	Methyl-b-d-glucopyranoside (MBdG)	+
Leucine arylamidase (LeuA)	-	Pullulan (PUL)	-
L-proline arylamidase (ProA)	-	D-raffinose (dRAF)	-
Beta glucuronidase (BGURr)	-	O/129 resistance (comp.vibrio.) (O129R)	-
Alpha-galactosidase (AGAL)	-	Salicin (SAL)	+
L-pyrrolydonyl-arylamidase (PyrA)	-	Saccharose/Sucrose (SAC)	+
Beta-glucuronidase (BGUR)	-	D-trehalose (dTRE)	+
Alanine arylamidase (AlaA)	+	Arginine dihydrolase 2 (ADH2s)	+
Tyrosine arylamidase (TyrA)	-	Optochin resistance (OPTO)	+
D-sorbitol (dSOR)	-	Urease (URE)	-
Polymixin b resistance (POLYB)	-		

### Phylogenetic analysis based on 16 S rRNA gene sequence

**Fig. 3:**
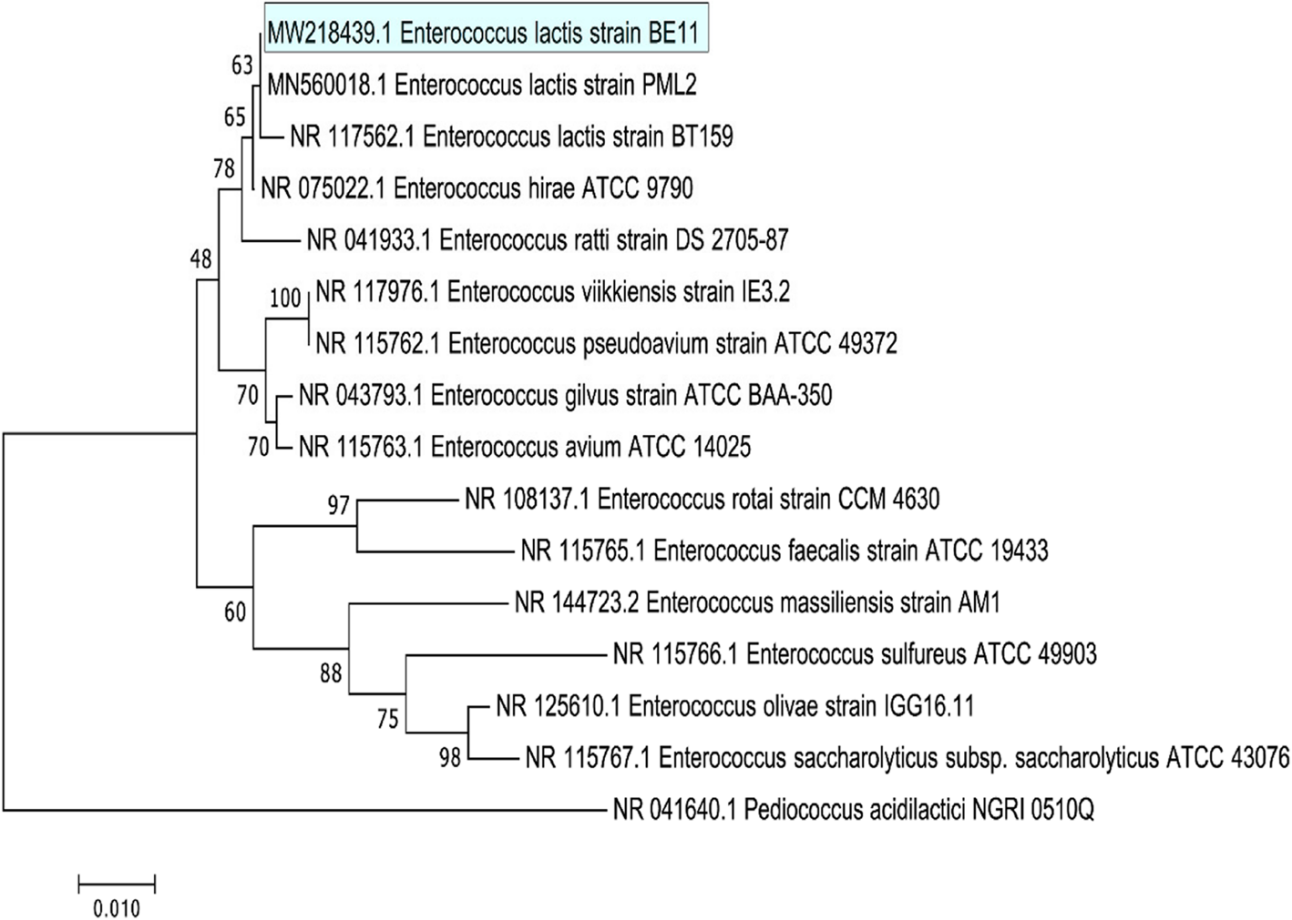
Phylogenetic tree based on the 16 S rRNA partial gene sequence of BE11 bacterial isolate identified as *Enterococcus lactis* by BLAST (NCBI) (submitted to GenBank under accession number MW218439.1). The tree is drawn to scale and branch lengths reflect the number of substitutions per site. The tree was generated using MEGA7 program [35]. *Pediococcus acidilactici* strain NGRI 0510Q (accession number NR 041640.1) was used as an outer group

The sequence of BE11 isolate 16 S rRNA gene was aligned against closely related *Enterococcus* species using the ClustalW program available through https://www.genome.jp/tools-bin/clustalw. The evolutionary relatedness between BE11 and *Entercoccus lactis* strain BML2 (NCBI accession No. MN560018.1) and *Enterococcus lactis* strain BT 159 (NCBI accession No. NR 117562.1) was demonstrated by phylogenetic analysis based on the Maximum Likelihood method [35] and bootstrapping (100 replicates). The three strains were placed in a monophyletic clade with 63% bootstrap confidence (Fig. 3).

### Total carbohydrate content (%)

The phenol-sulfuric acid method was used to determine the total carbohydrate in the purified polysaccharide. The percentage of carbohydrates was calculated based on the UV-VIS measurement which revealed a high carbohydrate content of ~ 87% using glucose as standard.

### Structural functionalization by FTIR

**Fig. 4:**
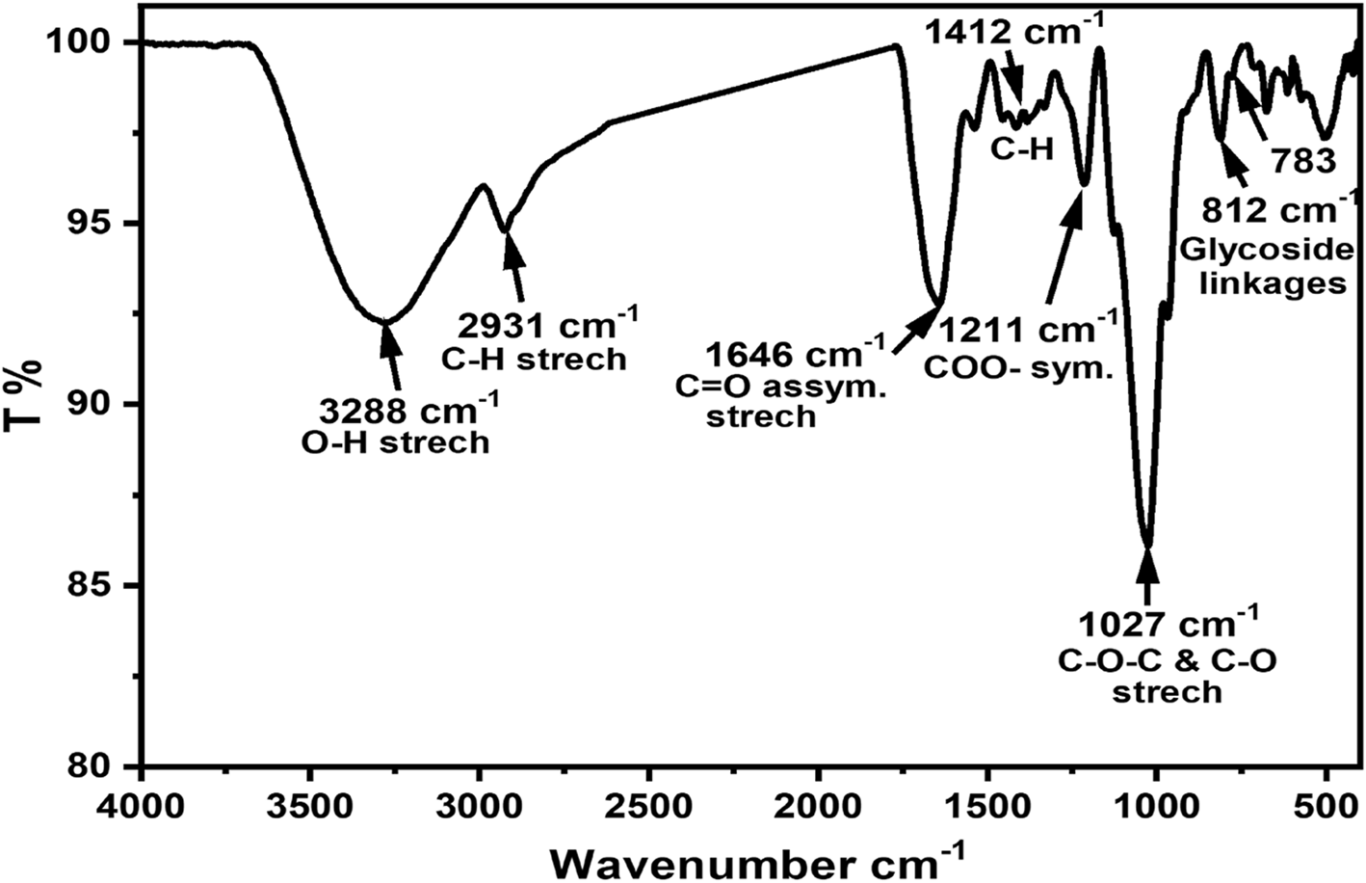
FTIR spectrum of the purified exopolysaccharide (BE11-EPS) produced by *Enterococcus* sp. BE11 isolate

The FTIR analysis reflected the characteristic functional groups of carbohydrates, Fig. 4. The signature of the IR spectrum is like previously reported polysaccharides, so the assignment of the IR bands was based on published data [46–48]. The IR spectrum revealed a strong broadband with the maximum at 3288 cm^−1^ corresponding to the stretching vibration of the hydroxy (O-H) group. The peak at 2931 cm^−1^ was assigned to the stretching vibration of the aliphatic C-H group, which is characteristic of polysaccharides.

The absorption bands at 1646 cm^−1^ belonging to the asymmetric stretching of the C = O group may be involved in H-bond [49]. The absorption region around 1400–1200 cm^−1^ was assigned to the stretching vibration of C = O of the carboxyl (COO^−^) group [50], and the flexural vibration of C-H within the polysaccharide’s structures [47]. In addition, the absorption region (1150–975 cm^−1^) with a maximum around 1027 cm^−1^ could be assigned to the stretching vibrations of the C-O and C-O-C groups [50, 51]. The shoulder at 1125 cm^−1^ indicated that the sugars exist mainly in the pyranose form [51]. The presence of the characteristic peaks at 812 cm^−1^ and 783 cm^−1^ referred to α- and β-glycoside linkages, respectively [52].

### Structural identification of BE11-EPS nuclear magnetic resonance (NMR)

**Fig. 5:**
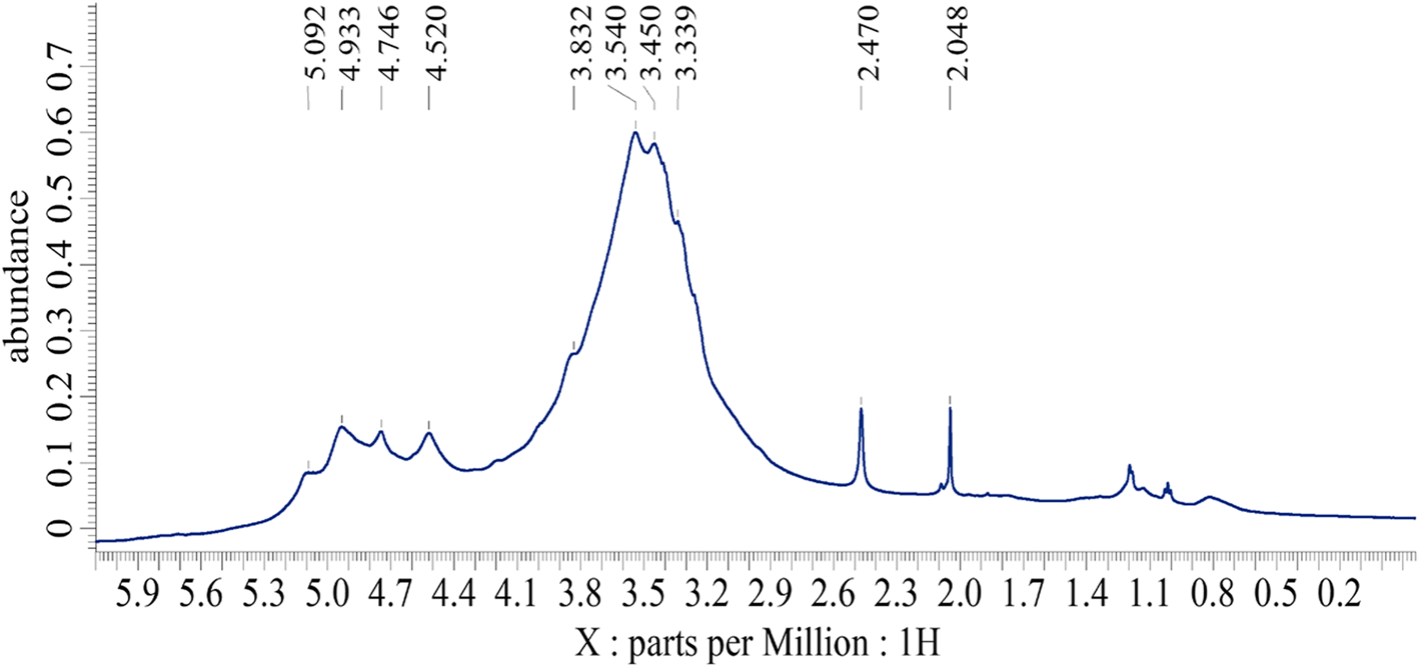
^1^H NMR spectrum of the exopolysaccharide (BE11-EPS) produced by *Enterococcus* sp. BE11 isolate recorded at 323 K (2% w/v; DMSO-*d*_6_, 500 MHz)

Preliminarily characterization of the purified BE11-EPS was carried out through the ^1^ H NMR experiment in DMSO-*d*_6_. The spectrum demonstrated broad signals with the characteristic signature of the polysaccharides, Fig. 5. Two regions were distinguished: (a) the up-field ring proton region in the range δ = 3.08–4.25 ppm related to the ring protons at positions C2 - C6 of the monosaccharide moieties, and (b) the downfield anomeric region appeared in the range δ = 4.45–5.12 ppm. The broad signal of H_2_O at 3.3 ppm (impurity in DMSO-*d*_6_) overlapped with the signals of the ring protons which hinders resolving. However, the anomeric region (δ = 4.45–5.12 ppm) highlighted the presence of monosaccharide residues of both types: β-anomeric sugars (δ = 4.45–4.75) and α-anomeric (δ = 4.84–5.12) sugars in the purified BE11-EPS compared to standard literature [53]. These results matched the FTIR analysis. Additionally, the broad signals of the anomeric protons caused difficulty to identify the mode of the glycosidic linkages (1 → 3, 1 → 4 and /or 1 → 6) between sugar moieties.

Furthermore, the presence of signals at 3.43–4.24 ppm in ^1^H NMR spectrum suggested the presence of galactose and glucose residues in the EPS backbone [54] which is in agreement with the GC-MS analysis. The low-intensity signal at δ = 1.19 was assigned to the proton chemical shift of the -CH_3_ of the L-fucose as reported in the literature [53] or L-rhamnose moiety with a low mol%, which was verified by GC-MS analysis.

### Monosaccharide composition by gas chromatography mass spectrometry

**Fig. 6:**
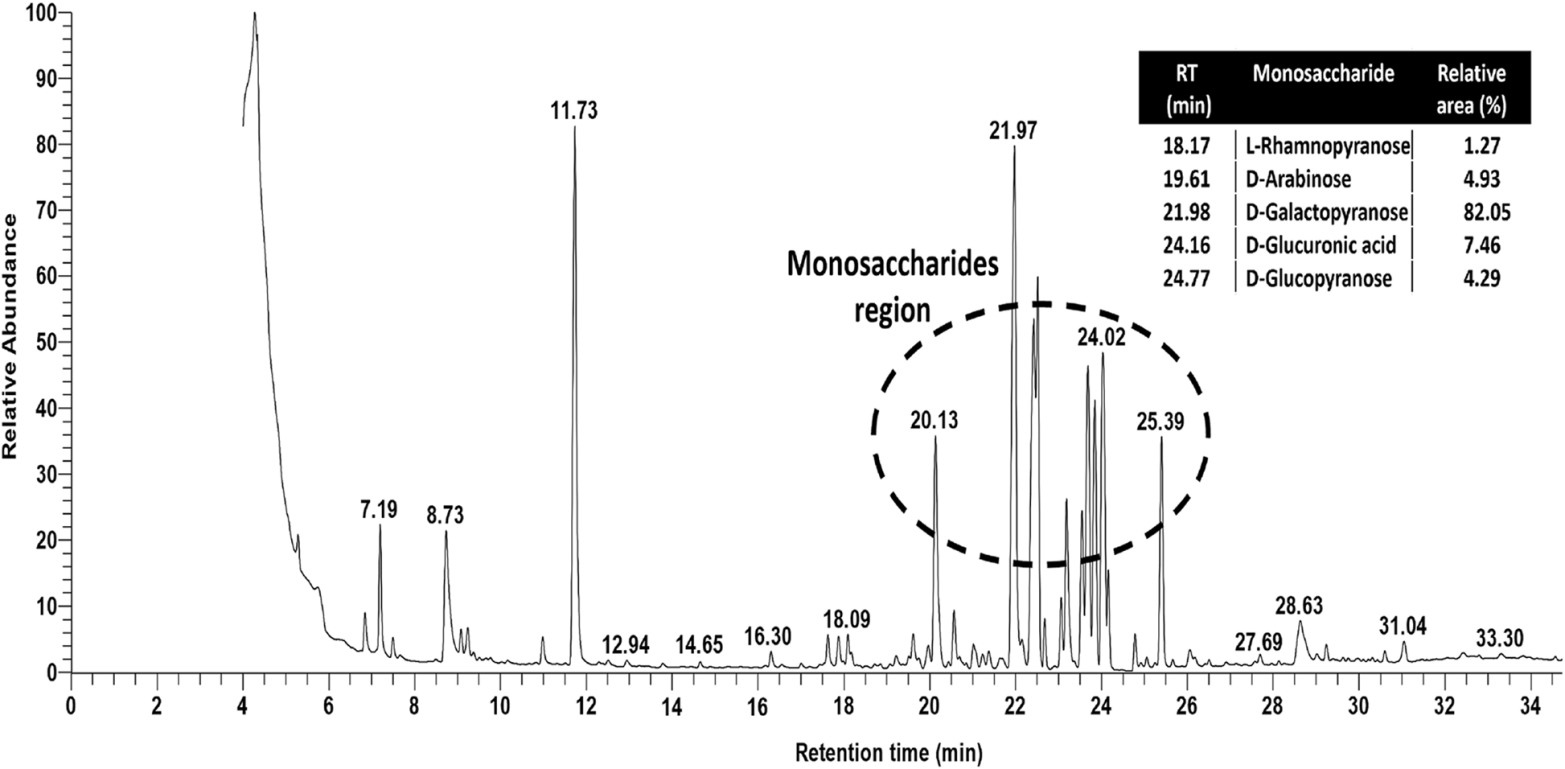
GC chromatogram of the purified exopolysaccharide (BE11-EPS) produced by *Enterococcus* sp. BE11 isolate

The monosaccharides forming BE11-EPS were investigated by the GC-MS analysis for the derivatized EPS sample. The GC chromatogram reflected intense peaks in the monosaccharide region (time = 18–25 min), with the identification of at least four different types of sugar, namely rhamnopyranose, arabinose, galactopyranose, glucopyranose and glucuronic acid, Fig. 6. Data analysis showed higher relative abundances area% from galactopyranose (82.0%) as the main constituent compared to the glucuronic acid (7.46), arabinose (4.93%), and glucopyranose (4.29%), while a smaller relative area% was determined for the sugar rhamnopyranose (1.27%).

### Morphological and elemental studies by SEM and EDX spectroscopy

**Fig. 7:**
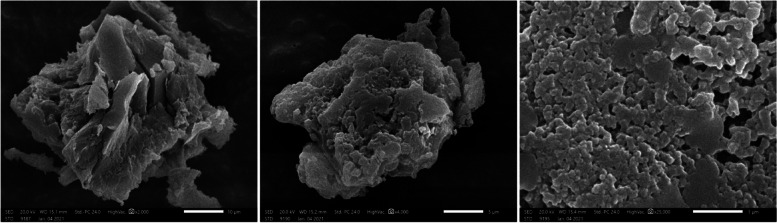
SEM micrographs of exopolysaccharide (BE11-EPS) produced by *Enterococcus* sp. BE11 isolate

**Fig. 8:**
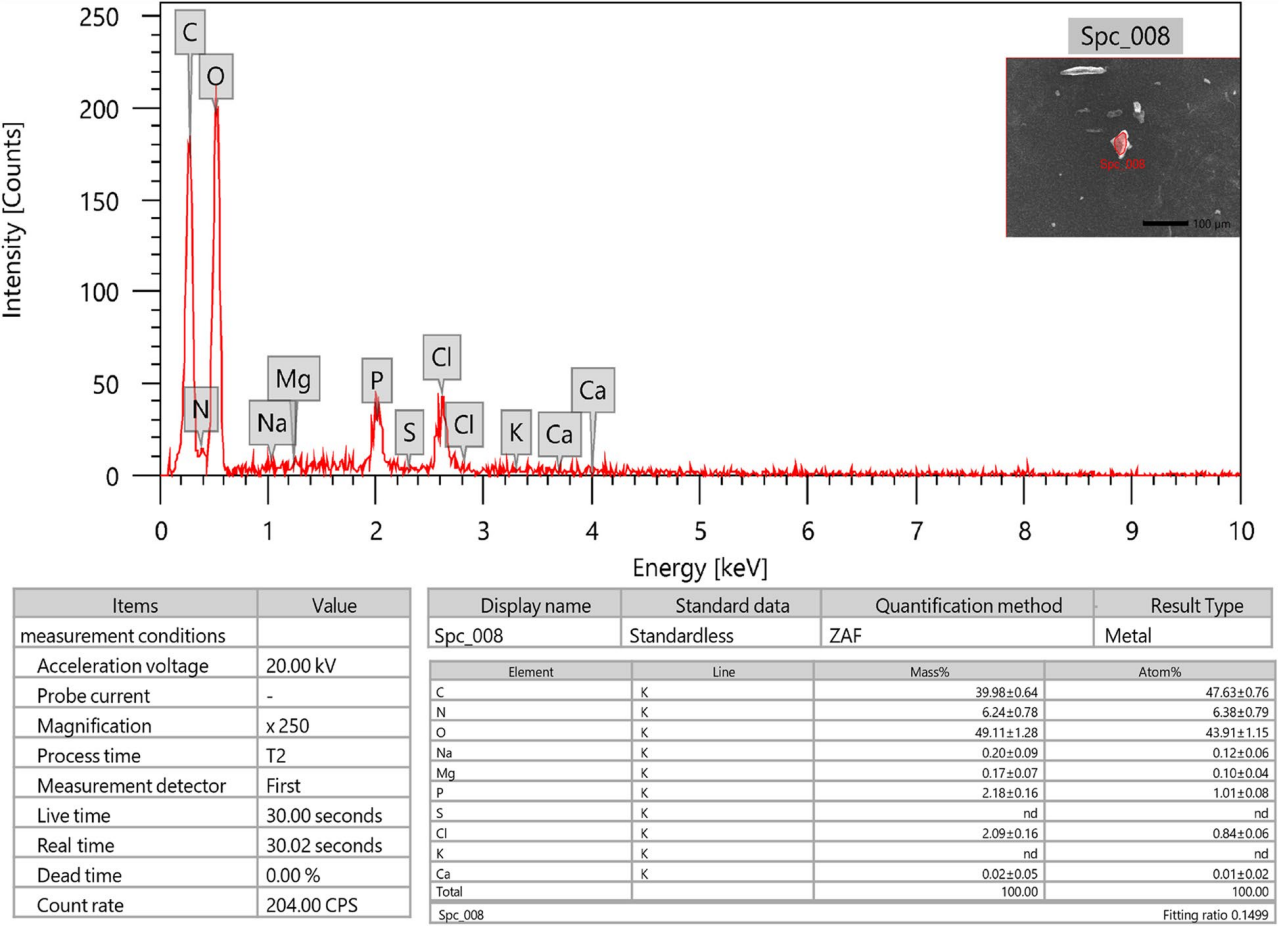
SEM-EDX data of the produced exopolysaccharide (BE11-EPS) produced by *Enterococcus* sp. BE11 isolate

The SEM micrographs of the purified BE11-EPS demonstrated amorphous texture and irregular surface morphology, Fig. 7. The EDX analysis showed that both carbon and oxygen are the major elements with mass ratios (w/w %) of 49.11 and 39.98%, respectively, suggesting the carbohydrate nature of the extracted product. The existence of heteroatoms such as nitrogen (6.24%) and phosphorus (2.18%), proposed traces of phospholipids and protein in the purified BE11-EPS, Fig. 8. Also, the analysis revealed traces of other elements including sodium, chloride, and magnesium, while no sulfur was detected.

### Anti-fish pathogens activity of BE11 culture cell-free supernatant and purified BE11-EPS

**Fig. 9:**
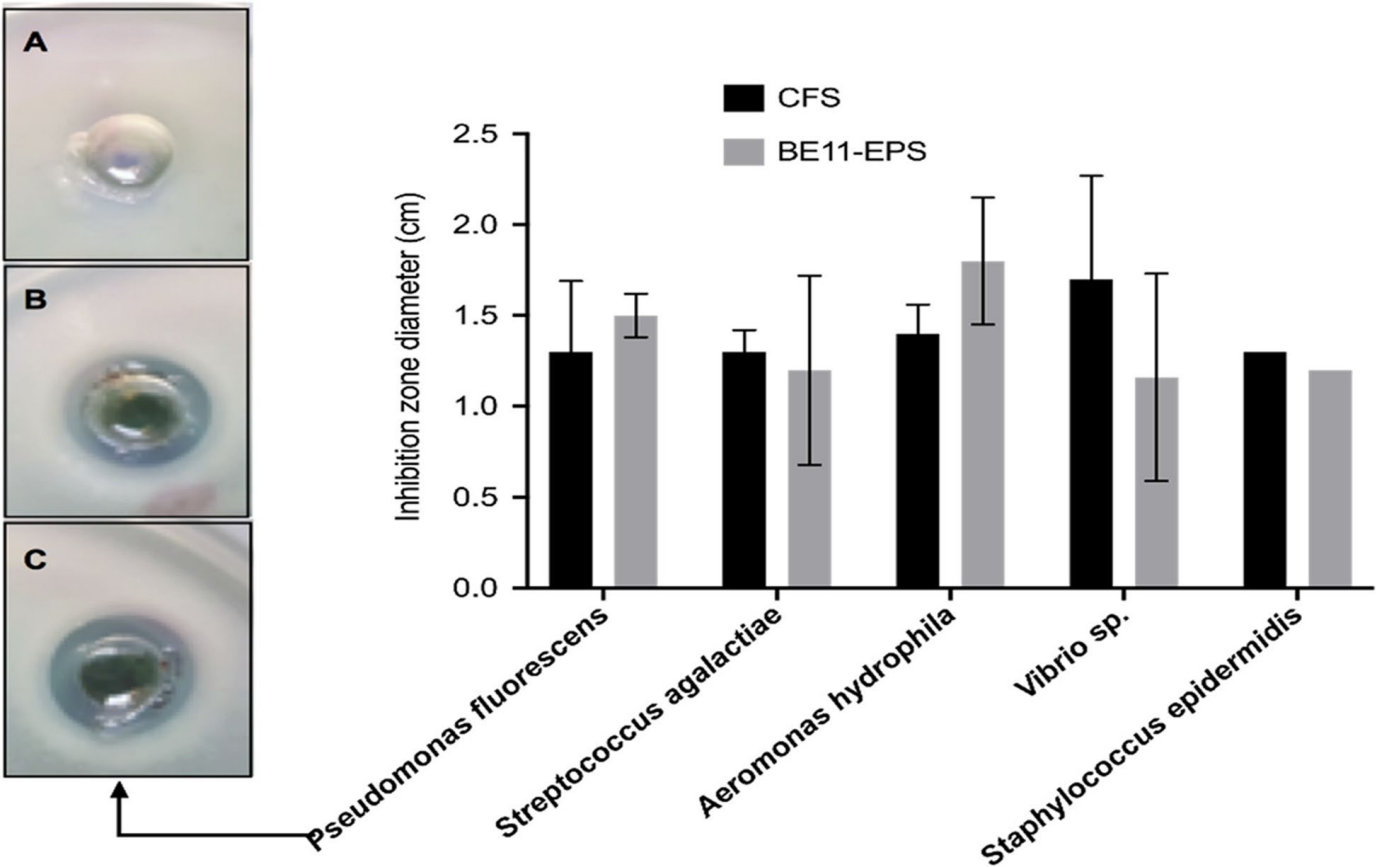
Measurements of inhibition zone diameter (cm) generated by either *Enterococcus* sp. BE11 isolate cell-free supernatant (CFS) (black columns) or purified exopolysaccharide (BE11-EPS) (grey columns) against the listed fish pathogens. The agar well-cut diffusion results for *Pseudomonas fluorescens* are demonstrated after exposure to dimethylsulfoxide (DMSO) (**A**), or to CFS (**B**) or BE11-EPS (**C**). DMSO served as a negative control well. Both CFS and BE11-EPS led to the inhibition of bacterial growth. The error bars refer to the standard deviation of three independent measurements

The antibacterial activity of BE11 cell-free supernatant (CFS) and purified BE11-EPS was tested against selected fish pathogens. The agar well-cut diffusion method demonstrated the promising anti-fish pathogens activity as demonstrated in Fig. 9. Specifically, CFS and BE11-EPS caused inhibition zones with all tested fish pathogens ranging from 1.3 to 1.7 and 1.2–1.8 (cm), respectively. The paired t-test of the inhibition zone diameter means (cm) between the CFS-treated and BE11-EPS-treated bacterial pathogens demonstrates a non-significant difference. This result implies that the effectiveness of the purified EPS against the tested pathogens is almost equivalent to the CFS.

## Discussion

The isolation of LAB from the intestinal tract of *Apis mellifera* has been reported in previous studies [55–57]. In general, the microbiota of honeybees is composed of yeasts, Gram-positive and Gram-negative bacteria. Examples of Gram-positive bacteria include *Bacillus* sp. *Lactobacillus* sp., *Enterococcus* sp., *Streptococcus* and *Clostridium*. Examples of Gram-negative bacteria include *Citrobacter*, *Enterobacter*, *E. coli*, *Pseudomonas*, and *Klebsiella* [57, 58]. This bacterial flora is considered endemic in the alimentary canal of adult bees. Interestingly, these associations do not depend on seasonal or food changes. The source of these normal flora comes from different sources, including contact with older bees and pollen consumption [55]. Moreover, the production of EPS by some bees associated *Enterococcus* sp. was reported in a recent study carried on honeybees live in Egyptian habitats [58]. However, scanning literature demonstrated that the EPS produced by honeybees’ gut microbial inhabitants was never chemically characterized. Therefore, the present study aimed to chemically characterize the EPS (BE11-EPS) produced by *Apis mellifera* gut-resident bacteria *Enterococcus* sp. isolate BE11. Furthermore, research was conducted to investigate the potential activity of this bacteria and its EPS as antibacterial agents against important fish pathogens and biofilm producers.

The basic characterization of the purified BE11-EPS indicated its high content of carbohydrates (~ 87%), which reflects the efficient extraction and purification methods. FTIR suggested that BE11-EPS was an acidic polysaccharide due to the broad absorption band in the region (3500–3100 cm^−1^), as well as the presence of bands in the region 1400–1200 cm^−1^ belonging to the symmetric and asymmetric stretching vibrations of the carboxylate (COO^−^) group. This assumption was consistent with GC-MS findings, which determined the glucuronic acid in the composition of the monosaccharides. Additionally, the GC-MS analysis classified the BE11-EPS of heterogenous nature made up of different monosaccharide moieties (galactose, glucose, arabinose, rhamnose, and glucuronic acid). NMR analysis ascertained the presence of the rhamnose through the signal at δ = 1.19 ppm, and most of the sugars existed in the pyranose form linked together through both α- and β-glycosidic bonds. Interestingly, the BE11-EPS revealed similar monosaccharides composition to the exopolysaccharide produced by the marine strain of *Rhodobacter johrii* CDR-SL 7Cii [59] and *Enterococcus faecium* WEFA23-2 [60] with different molar ratios.

Polysaccharides of different chemical structures, particularly the monosaccharides composition, may reflect various biological functions [61]. Xu et al. (2019) [62] reported two EPS from *Bacillus licheniformis* composed of monosaccharides namely mannose, ribose, glucuronic acid, galactose, arabinose, and fructose, while different mol%. The EPS exhibited potent antioxidant activity, besides inhibiting the α-amylase and α-glucosidase. Li et al., (2016) [63] studied a quantitative structure-activity relationship model of antioxidant activity of polysaccharides based on the contents of arabinose and galacturonic acid. They reported that galacturonic acid had the most remarkable role in the hydroxyl free radical scavenging activity of lentinan. In another study, four polysaccharides produced by *Laminaria japonica* were composed mainly of mannose, glucuronic acid, galactose, and fucose in different mol%. The authors reported that polysaccharides with higher uronic acid and sulfate contents revealed strong anticoagulant activities [64]. Li et al. (2018) [65] studied the structure characteristics, antitumor, immunomodulating, and gut-microbiota modulatory properties of polysaccharides from *Ganoderma lucidum* (GLW) and *Ganoderma sinense* (GSW). Both polysaccharides showed similar monosaccharides composition of mannose, glucose, and galactose; however, GSW showed a proportion of fucose. The polysaccharides exhibited similar tumor-suppressive activity in mice and possessed similar inducing effects to macrophages through inhibition against the viability and migration of cancer cells. Smiderle et al. (2011) [66] reported that the polysaccharide of *Agaricus bisporus* was mainly of mannogalactan, while the polysaccharide of *Agaricus blazei* showed higher content of β-glucan. They found that the extracts from both *Agaricus* species stimulated the production of pro-inflammatory cytokines and enzymes, while the polysaccharide extract of *Agaricus brazei* reduced the synthesis of these cytokines induced by LPS.

In the meantime, fish pathogens are dealt with in a variety of ways. Antibiotics are at the top of the list. Unfortunately, the world faces an antibiotic resistance dilemma caused by the overuse of antibiotics in aquatic systems. Antimicrobial resistance kills 700,000 people every year, with the figure expected to climb to 10 million by 2050 [27]. Therefore, it is vital to use novel antimicrobial drugs in aquacultures, particularly as one of the largest food production systems for humankind. Probiotics are an alternative to antimicrobial drugs; yet, probiotics, like other bacteria, are susceptible to antibiotic resistance genes acquisition by horizontal gene transfer. The existence of numerous antibiotic resistance genes in lactic acid bacteria from aquatic animals intended for use as probiotics in aquaculture was reported by Muñoz-Atienza et al. (2013) [67]. Therefore, recent trials were performed using non-living microbial cells or parts of the microbial cell instead of living probiotics. Hence, microbial exopolysaccharides produced by isolate BE11 may represent a promising alternative. Recent research reviewed in [68], verifies the antimicrobial activity of EPS against Gram-positive and Gram-negative bacteria, fungi, and viruses, as well as the antibiofilm capabilities of the EPS. For instance, EPS produced by other LAB isolated from other sources were reported to possess antibacterial, antifungal, and antiviral activities. For example, glucose-rich EPS produced by *Lactobacillus gasseri*, isolated from chicken, was found to possess antibacterial activity against some foodborne pathogens such as *E. coli* and *Staphylococcus aureus* [69]. Other galactose-rich EPS produced by *Lactobacillus rhamnosus* GG ATCC 53,103, isolated from a healthy human gastrointestinal microbiome, was found to exert antifungal activity against *Candida* sp. by interfering with its hypha formation and adhesion to epithelial cells [70]. Interestingly, sulfated EPS possessed antiviral activity [71].

Although there is no definitive mode of action to explain the observed antibacterial activity of BE11-EPS against the tested fish pathogens, yet there are many potential mechanisms. Sivasankar et al [54] reported the EPS ability to disrupt the bacterial cell wall peptidoglycan layer. Medrano et al [72] reported the masking nature of some bacterial EPS that interfere with receptors and channels on the surface of Gram-negative bacterial cells. Salachna et al [73] reported the association of EPS with secondary metabolites accumulation in the growth medium that adversely affect the growth of both Gram-positive and Gram-negative bacterial pathogens. On the other hand, the antimicrobial activity of isolate BE11 cell-free supernatant identified in this study may be attributed to the characteristic wide inhibitory effects of BE11 exopolysaccharide along with other metabolites reported in previous studies. For example, many LAB are known to produce lactic acid, ethanol, hydrogen peroxide, bacteriocins, and short volatile fatty acid chains [30, 74–76].

Finally, a mismatch between the biochemical and molecular identifications of bacterial isolate BE11 was observed. Other laboratories reported similar mismatches, for example, Moraes et al. (2013) reported a discrepancy between phenotypic and molecular approaches [77]. The authors tested the identification results of the Biolog system, API50, 16 S rRNA gene sequence, and species-specific PCR on 29 LAB isolates. The study concluded that a polyphasic approach is preferable for proper LAB identification, with molecular techniques being the most reliable. Most phenotypic identification system software databases are based on clinically significant bacteria, which could explain the disparity. In addition, phenotypic approaches suffer from poor reproducibility, as these systems are influenced by bacterial growth conditions, which affect gene expression patterns and thus test results. Therefore, using simple phenotypic techniques to identify bacterial cells may be misleading and is not suggested, particularly for bacterial isolates acquired from non-clinical sources such as food or bee gut. As a result, molecular approaches are thought to be more sensitive in these cases.

## Conclusion

The bee gut-resident *Enterococcus* sp. (BE11) and its purified BE11-EPS were found to possess promising antibacterial activity against important fish pathogens’ growth. The cell-free supernatant and BE11-EPS caused inhibition zones against all of the tested fish pathogens ranged from 1.3 to 1.7 and 1.2–1.8 (cm), respectively. This study represents the first characterization of EPS produced by an *Apis mellifera* gut microbial member. Future exploration of new sources of natural microbial EPS may contribute significantly to stop the spread of antibiotic resistance.

## Data Availability

All data generated or analyzed during this study are included in this article. The 16 S rRNA of *Enterococcus* sp. (BE11) generated in this study can be accessed through GenBank (NCBI) accession number MW218439.1.
